# Human herpesvirus 6A induces apoptosis of HSB-2 cells *via* a mitochondrion-related caspase pathway^☆^

**DOI:** 10.1016/S1674-8301(10)60059-0

**Published:** 2010-11

**Authors:** Lingyun Li, Jing Chi, Feng Zhou, Dandan Guo, Fang Wang, Genyan Liu, Chun Zhang, Kun Yao

**Affiliations:** a Department of Microbiology and Immunology, Nanjing Medical University, Nanjing, Jiangsu 210029, China; b Department of Developmental Genetics, Nanjing Medical University, Nanjing, Jiangsu 210029, China; c Department of Laboratory Medicine, the First Affiliated Hospital of Nanjing Medical University, Nanjing, Jiangsu 210029, China

**Keywords:** human herpesvirus 6, apoptosis, caspase, mitochondrion-mediated

## Abstract

Apoptosis plays an important role in the pathogenesis of viral infections. In this study, we investigated the cell death processes during productive HHV-6A infection and the underlying mechanisms. Annexin V-PI staining and electron microscopy indicated that HHV-6A is a strong inducer of apoptosis. HHV-6A infection decreased mitochondrial transmembrane potential and led to morphological changes of mitochondria. The cell death was associated with activation of caspase-3 and cleavage of DNA repair enzyme poly (ADP-ribose) polymerase, which is known to be an important substrate for activated caspase-3. Caspase-9 was activated significantly in HHV-6A-infected cells, whereas caspase-8 was not activated obviously. Moreover, HHV-6A infection upregulated Bax and downregulated Bcl-2. This is the first demonstration of mitochondrion-mediated, caspase-dependent apoptosis in HHV-6A-infected cells.

## INTRODUCTION

Apoptosis plays important roles in the development and homeostasis of cell populations and in the pathogenesis of disease[1],[2]. Apoptosis, a regulated suicide program, which is characterized by chromatin condensation, DNA fragmentation, membrane blebbing and cell shrinkage, can be induced by different factors such as virus infection, ultraviolet (UV) irradiation and deprivation of growth factors. Induction of apoptosis is mediated either through death receptors (extrinsic pathway), or at the mitochondrial level (intrinsic pathway)[3]–[5]. The intrinsic pathway involves activation of caspase-9 whereas the extrinsic pathway involves the activation of caspase-8, which is initiated by the interaction of Fas ligand with death receptors. Both caspase-8 and caspase-9 activate caspase-3, which, along with other caspases, cleave critical cellular proteins, resulting in apoptosis.

Human herpesvirus 6 (HHV-6) is a T-lymphotropic virus and the causal agent of exanthem subitum[6]–[8]. HHV-6 is involved in pneumonitis, hepatitis, mononucleosis-like illness and fatal hemophagocytic syndrome. HHV-6 remains latent after primary infection and reactivates in an immunocompromised state, as other human herpesviruses do. It has been observed that HHV-6 can infect various types of cells, although this virus mainly infects and replicates in CD4^+^ T-lymphocytes. HHV-6 has an immunosuppressive effect on T-cell functions, including IL-2 synthesis and cell proliferation[9],[10]. HHV-6 has been shown to induce apoptosis in cultured T cells, oligodendrocytes, astrocytes, neuronal cell lines, and CD4^+^ T lymphocytes isolated from healthy donors[11]–[14]. However, the molecular mechanisms by which HHV-6 induces apoptosis are not fully understood as yet.

In the present study, we investigated the effect of HHV-6A on apoptosis using human T-cell line HSB-2. We found that the mitochondrion-mediated apoptotic pathway was involved in HHV-6A-induced apoptosis. The cell death was associated with activation of caspase-3 and caspase-9. HHV-6A infection upregulated Bax, downregulated Bcl-2 and decreased mitochondrial transmembrane potential. To our knowledge, this is the first demonstration of mitochondrion-mediated, caspase-dependent apoptosis in HHV-6A-infected cells.

## MATERIALS AND METHODS

### Cells and viruses

Human T-cell line HSB-2 cells were cultured in RPMI 1640 medium containing 10% fetal calf serum (FCS). Cord blood mononuclear cells (CBMCs) were separated by Ficoll gradient centrifugation. The GS strain of HHV-6 variant A was inoculated into CBMCs, which were stimulated with 20 µg/mL phytohemagglutinin (PHA) for 2 d. Then, HHV-6A-infected CBMCs were cultured in RPMI 1640 with 10% FCS and 10 U/mL interleukin-2 (IL-2). When the cytopathic effects were maximal, the cells were lysed by freezing and thawing. Cell debris was removed by centrifugation at 1,000 *g* for 10 min. Virus was concentrated from the medium of infected cells by centrifugation at 80,000 *g* for 2 h. The pellets were suspended in a small volume of medium and used for infection. Titers were determined as viral DNA equivalents by quantitative PCR and confirmed by endpoint dilution of viral inocula on cell cultures. A multiplicity of infection (MOI) of 15 virus DNA copies per cell was used. Uninfected CBMCs were similarly cultured and treated as HHV-6-infected cells and used for mock infection. HSB-2 cells were either mock-infected or adsorbed with HHV-6 for 2 h at 37°C. After adsorption, the cells were incubated in growth medium at a concentration of 2.5×10^5^ cells/mL to allow optimal culturing devoid of cell stress due to excessive cell accumulation.

### Annexin V-propidium iodide (PI) staining

Apoptosis was measured using flow cytometry to quantify the levels of detectable phosphatidylserine on the outer membrane of apoptotic cells. Briefly, 5×10^5^ cells were collected, washed with PBS and resuspended in 500 µL binding buffer containing 10 mmol/L HEPES-NaOH (pH 7.4), 140 mmol/L NaCl, and 2.5 mmol/L CaCl_2_. Then, 5 µL of Annexin V-FITC (Bender MedSystems, Austria) and 5 µL of propidium iodide (PI) solution (Bender) were added and incubated in the dark for 15 min. The Annexin V-FITC and PI fluorescence were analyzed by flow cytometry. The amount of early apoptosis and late apoptosis was determined as the percentage of Annexin V^+^/PI– and Annexin V^+^/PI^+^ cells, respectively.

### Electron microscopy

Cells were fixed with 2.5% glutaraldehyde at room temperature for 1 h. After wash with PBS, the cells were collected, dehydrated in a series of 70%, 80% and 90% ethanol, and embedded in Epon. Ultrathin sections were cut and mounted on nickel grids and examined by transmission electron microscopy after staining with uranyl acetate and lead citrate.

### Determination of mitochondrial transmembrane potential (Δψm)

Mock-infected and HHV-6A-infected cells were collected and resuspended in 0.5 mL JC-1 incubation buffer (KeyGEN, China) at 37°C for 20 min in the dark. After incubation, the cells were washed twice with PBS and analyzed by flow cytometry. In healthy cells with high mitochondrial Δψm, JC-1 spontaneously forms complexes known as J-aggregates with intense red fluorescence. On the other hand, in apoptotic cells with low Δψm, JC-1 remains in the monomeric form, which shows green fluorescence.

### Analysis of activated caspase-3 by flow cytometry

The activation of caspase-3 in HHV-6A-infected HSB-2 cells was analyzed by flow cytometry with FITC-DEVD-FMK that recognizes cleaved caspase-3 according to the protocol provided by the manufacturer (Biovision Inc., USA). Briefly, mock-infected and HHV-6A-infected HSB-2 cells were collected and resuspended in 300 µL wash buffer, and 1 µL of FITC-DEVD-FMK was added and incubated for 1 h at 37°C. Cells were washed twice and analyzed by flow cytometry.

### Analysis of caspase-8 and caspase-9 using a colorimetric method

Caspase-8 and caspase-9 activities were determined using a colorimetric assay kit (KeyGEN). Briefly, mock-infected and HHV-6A-infected HSB-2 cells were collected and resuspended in 50 µL of lysis buffer and incubated on ice for 30 min. After centrifugation, the protein concentration was assayed by the BCA method, and 50 µg protein was diluted in 50 µL lysis buffer for each assay. Five µL of caspase-8, or caspase-9 substrate were added, respectively. The reaction mixtures were incubated at 37°C for 4 h. The released chromophore was measured at 405 nm using a microplate reader.

### Western blotting analysis

Whole cell extracts were prepared from cells by lysis in 1 mL lysis buffer containing 50 mmol/L Tris (pH7.4), 0.5% NP-40 and 0.01% SDS and a cocktail of protease inhibitors. Total protein (30 µg) was boiled for 5 min in 1× loading buffer, chilled on ice and then separated on 10% sodium dodecyl sulfate (SDS)-polyacrylamide gels. Subsequent to transfer onto PVDF membranes (Millipore, USA), non-specific protein interactions were blocked by incubation in 5% nonfat dry milk in TBST buffer containing 50 mmol/L Tris-HCl, 150 mmol/L NaCl, and 0.05% Tween 20 (pH7.6) at 4°C for 1 h. Membranes were then incubated at 4°C overnight with anti-PARP or anti-β-actin monoclonal antibody (Cell Signaling, USA), anti-Bcl-2 or anti-Bax (Bioworld, USA) in fresh blocking buffer. Unbound antibody was removed by three 10-min washes in TBST buffer. Membranes were then incubated with horseradish peroxide-conjugated secondary antibody (Santa Cruz Biotechnology, USA) for 1 h at room temperature followed by three 10-min washes with TBST buffer. The blot was developed with ECL reagent (Applygen Technologies, USA).

### Statistical analysis

Data were expressed as mean±SD. All statistical analyses were carried out using the SPSS software (Version 13.0). Comparison of variables between groups was performed by One-Way ANOVA followed by LSD post-hoc test. *P* values of <0.05 were statistically considered significant.

## RESULTS

### HHV-6A infection induces apoptosis of HSB-2 cells

**Fig. 1:**
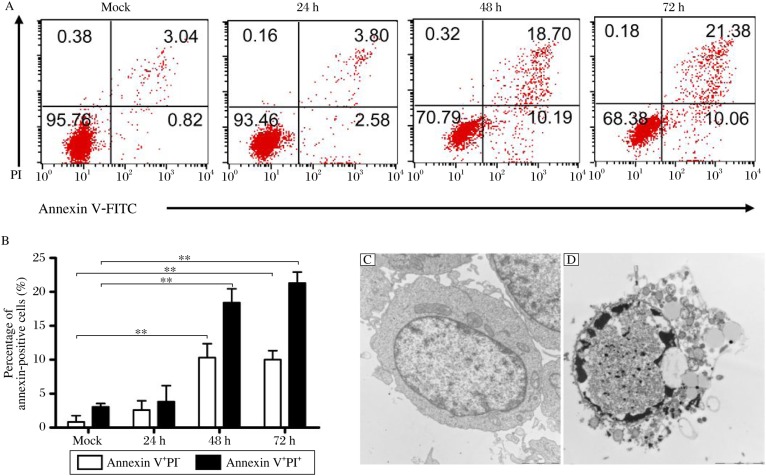
HHV-6A infection induces apoptosis of HSB-2 cells. A: HSB-2 cells were infected with HHV-6A for various times. Apoptosis was analyzed by flow cytometry. B: Each column and error bar represents the mean±SD of three independent experiments (**P < 0.01). Electron microscopic photographs of mock-infected cells (C) and HHV-6A-infected (D) cells. Mock cells (×12,000), infected cells at 72 h (×15,000). PI: propidium iodide.

To investigate the effect of HHV-6A infection on apoptosis in HSB-2 cells, we stained cells infected with HHV-6A with annexin-V-FITC and PI at 24, 48, and 72 h post-infection followed by analyzsis by flow cytometry. As shown in ***Fig. 1A*** and ***Fig. 1B***, a high percentage of annexin-V positive cells (apoptotic cells) in HHV-6A-infected cells was observed at 48 and 72 h compared to mock-infected cells were observed. The percentage of early apoptotic cells and late apoptotic cells reached 10.06% and 21.38% at 72 h post infection, whereas, in mock-infected cells, the percentage of early apoptotic cells and late apoptotic cells was only 0.82% and 3.04%, respectively. To further confirm the effect of HHV-6A infection on cell apoptosis, we examined the morphologic changes in HHV-6A-infected cells by transmission electron microscopy. HHV-6A-infected cells showed marginalized and condensed chromatin matrix, shrinkage and blebbing of the cytoplasm and fragmented nuclei, which are the typical features of apoptosis. In contrast, mock-infected cells showed uniformly stained nuclei (***Fig. 1C*** and ***Fig.1D***).

### HHV-6A infection disrupts the mitochondrial transmembrane potential (Δψm)

**Fig. 2:**
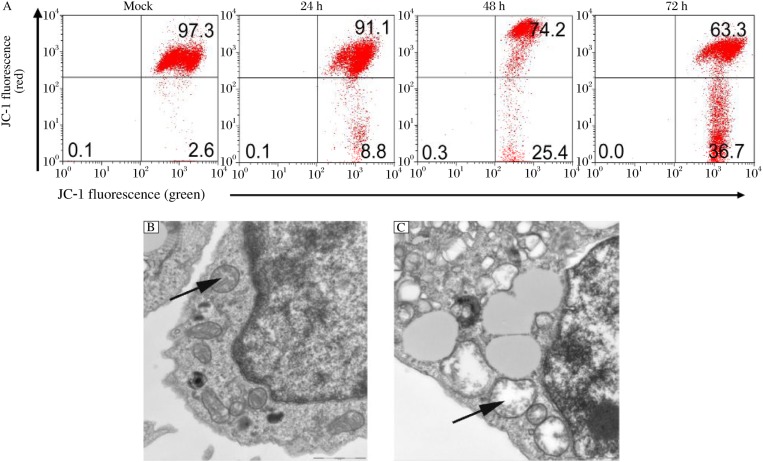
HHV-6A infection decreases mitochondrial transmembrane potential and changes mitochondrial morphology A: Mock-infected and HHV-6A-infected cells were stained with JC-1 and the mitochondrial transmembrane potential was measured by flow cytometry at 24, 48 and 72 h. Mitochondrial morphologies (arrow) of the mock-infected cells (B) and HHV-6A-infected cells (C) at 72 h were observed by electron microscopy.

The loss of mitochondrial transmembrane potential (Δψm) is an early event in cells undergoing mitochondrion-mediated apoptosis and has been observed in many virus-infected cells[15]–[19]. We examined the mitochondrial transmembrane potential in HHV-6A-infected HSB-2 cells with a cationic mitochondrion-specific dye, JC-1. As shown in ***Fig. 2A***, the percentage of cells with green fluorescence significantly increased from 2.6% (control) to 8.8%, 25.4% and 36.7% (HHV-6A-infected cells) at 24, 48 and 72 h post intection, respectively. These results indicated that HHV-6A infection induces Δψm collapse at the late stage of infection. In parallel with the Δψm changes, electron microscopic analysis also showed swelling and fewer convolutions in the cristae of the mitochondria in HHV-6A infected cells, whereas mitochondria remained intact in the mock-infected cells (***Fig. 2B***).

### HHV-6A infection triggers caspase-3 activation and PARP cleavage

**Fig. 3:**
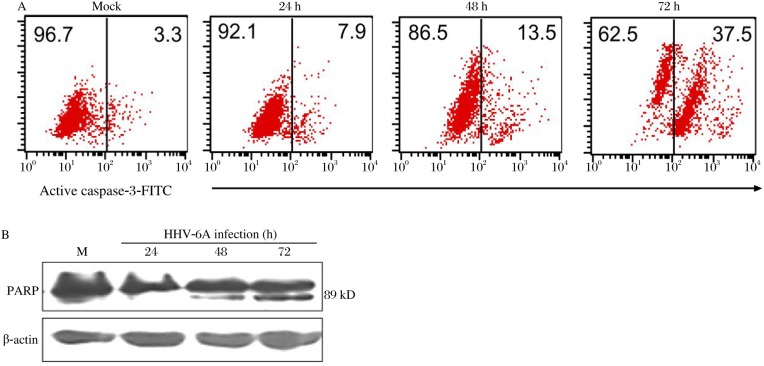
HHV-6A infection triggers caspase-3 activation and PARP cleavage in HSB-2 cells. A: Mock-infected and HHV-6A-infected cells were collected at various time points and the levels of activated caspase-3 were measured by flow cytometry. B: PARP in mock-infected and HHV-6A-infected HSB-2 cells was analyzed by Western blotting as described in Materials and Methods.

An important regulatory event in the apoptotic process is the activation of caspases, a family of cysteine proteases. Caspases are synthesized as inactive precursors (zymogens) that are processed to large and small subunits to form the active enzymes. Caspase-3 is one of the main effector caspases, which are activated in response to both intracellular and extracellular death signals. To explore the pathway by which HHV-6A induced apoptosis, we analyzed caspase-3 activity in HHV-6A-infected HSB-2 cells with anti-active caspase-3 antibody by flow cytometry. As shown in ***Fig. 3A***, HSB-2 cells with activated caspase-3 was about 7.9%, 13.5% and 37.5% at 24, 48 and 72 h, respectively, whereas the value was only 3.3% in the mock-infected cells.

PARP is an established substrate for caspase-3 in the apoptotic events. Cleavage of PARP facilitates cellular disassembly and serves as a marker of cells undergoing apoptosis[20]. We performed Western blotting to detect endogenous full-length PARP (116 kD), as well as the large fragment (89 kD) of PARP resulting from caspase cleavage. As shown in ***Fig. 3B***, the 89 kD cleaved fragment of PARP was detected in HHV-6A-infected cells at 48 and 72 h, but was not detected in the mock-infected cells.

### HHV-6A infection induces caspase-9 activation

**Fig. 4:**
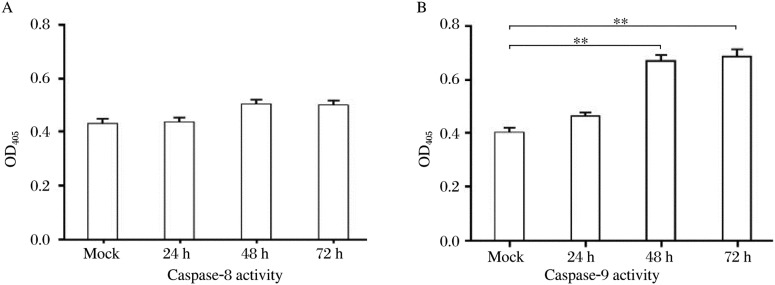
HHV-6A infection induces caspase-9 activation but not caspase-8. The activation of caspase-8 (A) and caspase-9 (B) was examined by colorimetric method using lysates from mock-infected and HHV-6A-infected HSB-2 cells. Each column and error bar represents the mean±SD of three independent experiments (**P < 0.01).

Apoptosis proceeds mainly by two pathways, the extrinsic pathway triggered by receptor activation leading to caspase-8 activation and the intrinsic pathway characterized by the release of cytochrome C that results in the activation of caspase-9. To further define whether HHV-6A induces apoptosis *via* the intrinsic or the extrinsic pathway, we measured the activities of caspase-8 and -9, respectively. As shown in ***Fig. 4***, infection with HHV-6A resulted in a 1.7 fold increase in caspase-9 activities at 72 h compared with controls. However, there were no significant changes of caspase-8 activities in HHV-6A-infected cells compared with those of mock-infected cells at 24 to 72 h. These data suggested that HHV-6A induces apoptosis in HSB-2 cells by the intrinsic pathway.

### HHV-6A infection increases the Bax/Bcl-2 ratio

**Fig. 5:**
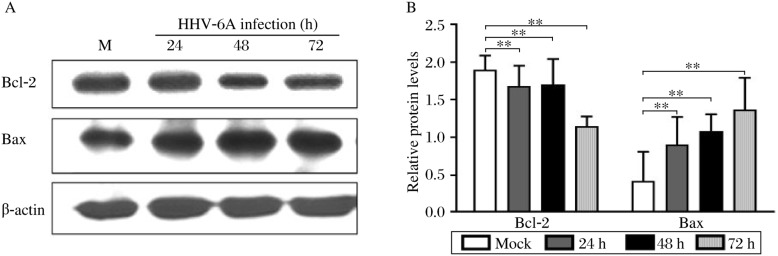
HHV-6A infection decreases Bcl-2 and increases Bax expression. A: Expressions of Bcl-2 and Bax were detected by Western blotting using anti-Bcl-2 and anti-Bax antibodies, respectively. β-actin was used as a loading control. B: Quantitative values of Bcl-2 and Bax are the mean from three independent experiments (**P < 0.01).

The mitochondrial intrinsic pathway of apoptosis is regulated by the balance between the pro- and antiapoptotic Bcl-2 family of proteins[21],[22]. The proteins of the Bcl-2 family are known to directly regulate mitochondrial membrane permeability[23]. We performed immunoblotting analyses to determine whether the effects of HHV-6A infection on cell apoptosis were associated with changes of antiapoptotic (Bcl-2) and proapoptotic (Bax) proteins. As shown in ***Fig. 5***, the levels of Bcl-2 protein were significantly decreased following HHV-6A infection compared to that in mock-infected cells, whereas the expression of Bax protein was significantly increased in HHV-6A-infected cells. These results indicated that HHV-6A infection in HSB-2 cells could provoke cell death by both suppression of anti-apoptosis gene expression and promotion of pro-apoptosis gene expression.

## DISCUSSION

HHV-6 is a member of the *Betaherpesvirinae* subfamily. Two major subgroups with distinct genetic, immunological and virologic characteristics have been named as HHV-6A and HHV-6B[24]–[28]. Ichimi *et al.* reported that in cord blood lymphocytes, HHV-6 induced apoptosis by an unknown mechanism, which was independent of death receptors[29]. In contrast, Inoue *et al.* reported that both TNF-α and anti-CD95 and anti-CD95L antibodies augmented HHV-6-induced apoptosis, suggesting an involvement of death receptors in HHV-6-induced apoptosis[11]. Caspases play a critical role in apoptosis. During apoptosis, caspases cleave specific substrates and activate downstream molecules, which eventually culminate in cell death[30]. Although HHV-6-induced apoptosis has been widely confirmed, the role of the caspase cascade in HHV-6-induced apoptosis has not been studied. In this study, we demonstrated that activated caspase-9 and caspase-3 were increased in HHV-6A-induced apoptosis of T cells, but there were no significantly changes of activated caspase-8. In addition, we found that PARP was cleaved in HHV-6A-induced T cells apoptosis. Caspase-3 is a common effector of both death receptor and the mitochondrial signaling pathways. Caspase-8 is activated by the death receptor signaling pathway, whereas caspase-9 is activated in the mitochondrial signaling pathway during apoptosis. We speculate that HHV-6A-induced apoptosis could be *via* the mitochondrial apoptotic pathways.

It was reported that simian varicella virus induces apoptosis in monkey kidney cells by the intrinsic pathway and involves the downregulation of Bcl-2 expression[31]. Bax was activated during hepatitis C virus and rotavirus-induced apoptosis through the mitochondrial pathway[17],[32]. Since Bcl-2 family proteins are central regulators of the mitochondrial apoptotic pathway and have been implicated in various models of virus-induced apoptosis[17],[33]–[34], we examined the expression of Bcl-2/Bax in HHV-6A-induced mitochondrial dysfunction. Our data showed that the antiapoptotic protein Bcl-2 decreased, which was accompanied by the increase of proapoptotic protein Bax during HHV-6A infection, suggesting that Bcl-2 and Bax were involved in the apoptosis of HHV-6A-infected T cells.

Virus-induced cell death is a complex and important aspect of the pathogenesis of virus infection[35]. In recent years, many viruses and viral gene products in different families have been found to induce apoptosis during their infection cycles[36]–[38]. Apoptosis may facilitate the release of virus progeny and help the virus to evade the immune surveillance by attenuating inflammation. On the other hand, premature apoptosis, most likely evoked by host defense mechanisms, aborts virus infection and therefore limits virus productivity and infectivity. The intricate balance between life and death of infected cells must be regulated by viral products or by interaction between virus and host to ensure a successful infection cycle.

In summary, we demonstrated that HHV-6A induces cell apoptosis in cultured T lymphocyte cells through the mitochondria-mediated apoptosis pathway, as evidenced by (1) decreasing the mitochondrial transmembrane potential and promoting mitochondrial swelling; (2) activation of caspase-9; (3) decreasing the expression of anti-apoptotic Bcl-2 protein and increasing the expression of proapoptotic Bax protein. The identification of the apoptotic signaling pathways in HHV-6 infected cells would be helpful in understanding the mechanisms by which HHV-6 infection causes functional alterations of host cells.
